# Post-kala-azar dermal leishmaniasis in the Indian subcontinent: A threat to the South-East Asia Region Kala-azar Elimination Programme.

**DOI:** 10.1371/journal.pntd.0005877

**Published:** 2017-11-16

**Authors:** Eduard E. Zijlstra, Fabiana Alves, Suman Rijal, Byron Arana, Jorge Alvar

**Affiliations:** 1 Drugs for Neglected Diseases initiative, Geneva, Switzerland; 2 Rotterdam Centre for Tropical Medicine, Rotterdam, the Netherlands; 3 Drugs for Neglected Diseases initiative, India Office, New Delhi, India; Pasteur Institute of Iran, ISLAMIC REPUBLIC OF IRAN

## Abstract

**Background:**

The South-East Asia Region Kala-azar Elimination Programme (KAEP) is expected to enter the consolidation phase in 2017, which focuses on case detection, vector control, and identifying potential sources of infection. Post-kala-azar dermal leishmaniasis (PKDL) is thought to play a role in the recurrence of visceral leishmaniasis (VL)/kala-azar outbreaks, and control of PKDL is among the priorities of the KAEP.

**Methodology and principal finding:**

We reviewed the literature with regard to PKDL in Asia and interpreted the findings in relation to current intervention methods in the KAEP in order to make recommendations. There is a considerable knowledge gap regarding the pathophysiology of VL and PKDL, especially the underlying immune responses. Risk factors (of which previous VL treatments may be most important) are poorly understood and need to be better defined. The role of PKDL patients in transmission is largely unknown, and there is insufficient information about the importance of duration, distribution and severity of the rash, time of onset, and self-healing. Current intervention methods focus on active case detection and treatment of all PKDL cases with miltefosine while there is increasing drug resistance. The prevention of PKDL by improved VL treatment currently receives insufficient attention.

**Conclusion and significance:**

PKDL is a heterogeneous and dynamic condition, and patients differ with regard to time of onset after VL, chronicity, and distribution and appearance of the rash, as well as immune responses (including tendency to self-heal), all of which may vary over time. It is essential to fully describe the pathophysiology in order to make informed decisions on the most cost-effective approach. Emphasis should be on early detection of those who contribute to transmission and those who are in need of treatment, for whom short-course, effective, and safe drug regimens should be available. The prevention of PKDL should be emphasised by innovative and improved treatment for VL, which may include immunomodulation.

## Introduction

Kala-azar/visceral leishmaniasis (VL) is endemic in the Indian subcontinent (ISC), affecting the Gangetic plains of India, Bangladesh, and Nepal.

ln 2005, the governments of Bangladesh, India, and Nepal agreed on a regional initiative, the Regional Kala-azar Elimination Programme (KAEP), to eliminate VL as a public health problem from the region by 2015, which has now been extended to 2017 [1]. The factors favourable for the elimination of kala-azar in this region included high endemicity, limited to 1 geographical region in the 3 countries (45 districts in Bangladesh, 52 in India, and 12 in Nepal); the absence of an animal reservoir; *Phlebotomus argentipes* being the only vector and susceptibility to insecticide; the availability of an accurate rapid diagnostic test (RDT) detecting antibodies against the 39-aminoacid-recombinant kinesin antigen (anti rK39); the use of an oral drug, miltefosine; and strong political commitment from the 3 countries [2]. Subsequently, other efficacious treatments, including combination therapies and single dose liposomal amphotericin B (AmBisome), which replaced miltefosine, became available. The key strategies for elimination were early diagnosis and complete case management; integrated vector management and vector surveillance; effective disease surveillance through passive and active case detection; social mobilisation and building partnerships; and implementation and operational research [3].

This multidisciplinary approach strategy aimed at reaching the elimination target of less than 1 kala-azar case in 10,000 population by 2015. Current figures are promising, with a substantial decline in cases by 59% and a reduction of mortality by 85%. Nepal has eliminated the disease at the district level and sustained the situation for the past 2 years, Bangladesh has achieved the elimination target in more than 90% of endemic districts (upazilas), and India has achieved the target in more than 70% of endemic districts (blocks) [3].

From the attack phase, the KAEP is moving to the consolidation phase, aiming to identify (new) low-endemic foci by active case detection, consolidating vector control measures, and treating potential sources of infection, of which post-kala-azar dermal leishmaniasis (PKDL) is the most important [4,5].

PKDL is a dermal condition that in Asia occurs in a significant percentage (10%–20%) of patients after VL treatment [6]. PKDL consists of painless macular or papulonodular (PN) lesions—or a mix of both—that harbour parasites that may be exposed to the bite of sand flies, thus possibly playing a major role in the transmission cycle [7,8].

PKDL is therefore recognised as a constraint in the elimination effort, and the development of strategies for case finding, diagnosis, and treatment is among the objectives in the KAEP [4].

These strategies are currently not based on solid scientific evidence, with costly case finding, risk of misdiagnosis, and risk of inadequate and unnecessary treatment with potentially toxic drugs as a result.

In this paper, we review the available information on PKDL relevant for the KAEP, identify essential gaps in knowledge, and make recommendations.

## Search strategy and selection criteria

We searched the electronic database PubMed for articles published from January 1900 to May 2017 by use of the terms ‘PKDL,’ ‘post-kala-azar dermal leishmaniasis,’ ‘kala-azar,’ ‘visceral leishmaniasis,’ and ‘VL’ paired with the terms ‘India,' ‘Bangladesh,’ ‘Nepal,’ or ‘Indian subcontinent’ in the English language. Relevant articles from the authors’ personal files were identified. From the articles, certain relevant references were searched and included.

## Epidemiology

### PKDL rate and prevalence of PKDL

The most recent studies (published between 2000 and 2017) are summarised in Table 1.

**Table 1:** Summary of epidemiological studies published from 2000 to 2017 on prevalence, incidence, and interval between onset of PKDL and VL treatment.^a^.

Country	Region	Year	Type of study	Case finding	Prevalence	Incidence	PKDL rate	Interval after VL	Ref.
India	Bihar	2012	Community based	active	4.4/10,000 population (confirmed cases) 7.8/10,000 population (probable cases)			median: 30 months range: 9–129 months	[9]
	West Bengal	2012	Community based	active	25/2,435 population point prevalence 1%			mean: 3.13 years range: 6 months to 14 years <1 year: 3 of 25	[10]
	Patna	2007–2012	Hospital based	passive			25 PKDL/8,311 VL cases treated (0.3%)	median: 1.2 years (IQR 0.8–2.2) <12 months: 36%	[11]
	Patna	not recorded	Hospital-based case series (*n* = 60)	passive				3 months to 10 years	[12]
	New Delhi	1995–2014	Hospital-based case series (*n* = 282)	passive				<12 months: 13% 1–5 years: 56% >5 years: 31%	[13]
Bangladesh	Fulbaria	2007–2008	Community based	active		1/10,000 PY (2002–2004) 21/10,000 PY (2007)	9.9%	median: 21 months (95% CI 16.4–25.6)	[14]
	Fulbaria	2010–2013	Community based	active			12-month FU 0.6% 24-month FU 8.1% 36-month FU 10.2%	range: 0–36 months	^b^
	Fulbaria	2007–2010	Community based	active			12-month FU 3% 24-month FU 10% 60-month FU 17%	median: 19 months range: 0–120 months	[15]
	Trishal	2010	Community based	active	6.2/10,000 population			median: 36 months (IQR 24–48)	[16]
	Fulbaria	2007	Community based	active	3.2–7.3/1,000 population in 4 paras		13.9 (entire community); 16% in 4 of 9 paras	6–24 months: 40% 0–6 months: 20%	[17]
Nepal	Endemic districts	2000–2009	Retrospective cohort	active			mean 2.4% <2 years: 1.4% <8 years: 3.6%	23 months (IQR 15–41)	[18]
	Dharan	1998–2000	Hospital-based case series (*n* = 22)	passive				mean: 26.9 ± 11.9 months range: 6–60 months	[19]

^a^Note differences in methodology used: field-based versus hospital-based studies; active versus passive case finding; measurement of prevalence versus incidence; and confirmed versus probable PKDL cases.

^b^Personal communication, Koert Ritmeijer, Médecins sans Frontières, to Eduard Zijlstra.

Abbreviations: FU, follow-up; IQR, interquartile range; PKDL, post-kala-azar dermal leishmaniasis; PY, person-year; VL, visceral leishmaniasis.

In India, the exact PKDL rate (i.e., the frequency with which PKDL follows after treatment of VL) is not known exactly because cohort studies with active follow-up of VL cases are not available. Passive case finding yielded a PKDL rate of 0.3% [11].

In Bangladesh, 2 prospective studies showed that PKDL rates increased with longer duration of follow-up: in 1 study, the rate was 10.2% by 3 years and 17% by 5 years, respectively (personal communication, Koert Ritmeijer, Médecins sans Frontières [MSF] Holland, to Eduard Zijlstra). Another study showed increased prevalence from 1 case to 21 cases per 10,000 person-years in 2002–2004 and 2007, respectively [15].

### Interval after VL

In contrast to earlier studies in which the interval between treatment of VL and onset of PKDL was thought to be at least 1–3 years or longer, recent information shows that PKDL may occur much earlier, with up to 36% of cases presenting within 1 year after VL [13,17,20,21]. This is more in agreement with the experience in Sudan (which has the highest global PKDL rate, up to 60%), where the interval after VL is 0–13 months [22].

This interval is influenced by previous treatment for VL; in India, in those previously treated with sodium stibogluconate (SSG), the interval was longer as compared with those treated with AmBisome (2.9 years and 1.2 years, respectively) [11]. In another study, the median interval after VL treatment with SSG was 36 months, while this was 48 months and 21 months for those treated for VL with amphotericin deoxycholate or miltefosine, respectively [13].

Age is also important because younger patients (<15 years) were reported to have significantly shorter intervals than the older age group: median intervals were 26 months and 50 months, respectively [16].

Concomitant VL and PKDL, often referred to as para–kala-azar dermal leishmaniasis, has only sporadically been reported in the ISC, while this seems more common in Sudan (up to 18%) [15,23,24].

In 10%–23% of cases, there is no history of previous VL; this is similar to what has been reported from Africa [10,13,14,20,25,26].

### Gender balance

While in hospital-based studies in nonendemic areas a male preponderance (80%) is reported among PKDL patients with a male-to-female ratio of 3–4:1, in endemic areas, the ratio seems much more balanced (ratio 1.2–1.9) and not different from that found in VL [10–12,14,27–29].

### Active case finding

Because PKDL patients are not ill and the disease may run a chronic course with limited discomfort, many do not report to health facilities or report late. Only up to 25% of patients seek medical treatment for cosmetic reasons. This is concerning because of the possible risk in transmission [18,30,31]. Various strategies have been used for active case detection [10,16,29,32,33].

Using a cluster approach in following up treated VL patients, active case detection was shown to be superior to passive case detection in Bangladesh (Fulbaria and Trishal districts); for PKDL, the yield increased 30-fold, while for VL, this was 1.4-fold, emphasising the lack of urgency felt by PKDL patients to seek medical care. However, in both strategies, patients had already been symptomatic for 345–360 days prior to diagnosis (clinically and by a positive rK39 RDT) [29].

### PKDL and transmission

**Fig 1:**
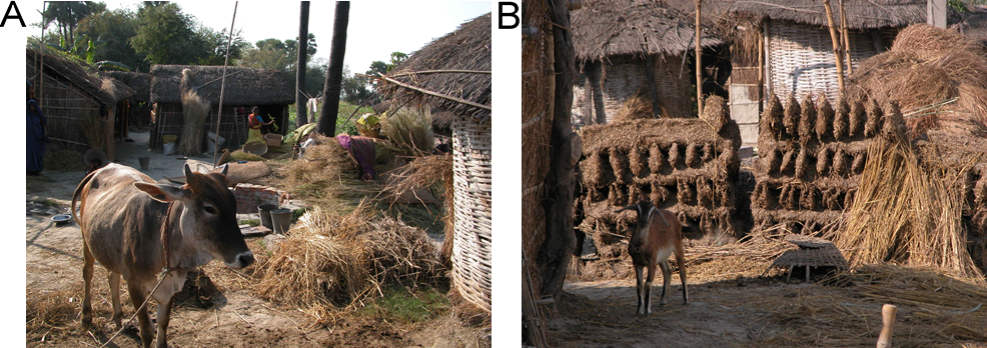
Endemic area for VL in India (Muzaffarpur). (A) People live in close contact with animals that may attract sand flies. (B) Typical houses with walls made of mud. VL, visceral leishmaniasis.

Studies have indicated that *P*. *argentipes* is the sole vector of *Leishmania donovani* in the ISC [34]. It also feeds on animals, mainly cattle, that are not a reservoir for parasites but whose presence influences biting behaviour and breeding sites and thus, indirectly, transmission (Fig 1) [35]. Other animals, including goats, have been suggested as reservoirs, but there is no conclusive evidence as to their role in transmission [36]. After parasites had been demonstrated in sand flies feeding on 2 patients with nodular lesions in a study from 1933, a 30% infection rate of sand flies was reported after feeding on a patient with depigmented (macular) PKDL lesions (the predominant type in the ISC), in which parasites are normally scanty in a slit-skin smear or biopsy [37]. In a later experiment, 53% of sand flies that fed on 4 PKDL patients with nodular or nodulo-ulcerative lesions developed infection; this was thought to be an explanation for a recent VL outbreak in southern West Bengal in the 1980s [7]. These experiments also showed that after feeding on a PKDL patient, the parasite developed in the sand fly in the same way as after feeding on a VL patient and that the strain is capable of causing visceralised disease in mice and hamsters [38]. Recently, 3 PKDL patients from Bangladesh were found infective to sand flies, including a patient with macular lesions. [39]

Despite this, in a longitudinal study over 2 years in Bihar, a highly endemic area, no cases of VL were observed in households with PKDL. Because the immune status of participants was not assessed but likely to be high, it may be argued that different results could have been obtained in an interepidemic period [26].

### Epidemiological modelling

Various models have attempted to describe and quantify the influence of PKDL in transmission of VL in the ISC. Dye (1992) based his model on data collected between 1875 and 1950 in Assam, where, from 1920, cases were treated with tartar emetic and the PKDL rate was 25%. The model predicted that if 0.5% of PKDL cases remained infectious, an epidemic may become endemic [40].

Stauch et al. (2011) based their VL model in the ISC on data from the KALANET trial and assumed a PKDL rate of 3% and a prevalence of 0.5 cases/10,000 [41]. Active case finding and effective short-course treatment of PKDL were recommended. Recently, similar recommendations were made, and the need to determine and quantify the infectivity of PKDL was emphasised because this may influence the impact of insecticide residual spraying (IRS) [42,43].

## Pathogenesis

It is likely that PKDL results from the persistence of parasites from VL in the skin, leading to a block in the development of an adequate immune response. Using the (imperfect) helper T cell 2 (Th2)/helper T cell 1 (Th1) dichotomy, the immune response in African PKDL has characteristics of a Th2 response in the skin, while systemically, the Th1 response predominates, the latter being the result of successful VL therapy [44,45]. Alternatively, a new infection by leishmania parasites in an individual who has already acquired a degree of immunity has been suggested [46].

Recently, an association was found, which needs further study, between chronic environmental arsenic exposure and the development of PKDL [47].

The main factors in the pathogenesis of PKDL, including host- and parasite-related factors, are summarised in S1 Table.

## Clinical features and diagnosis

**Fig 2:**
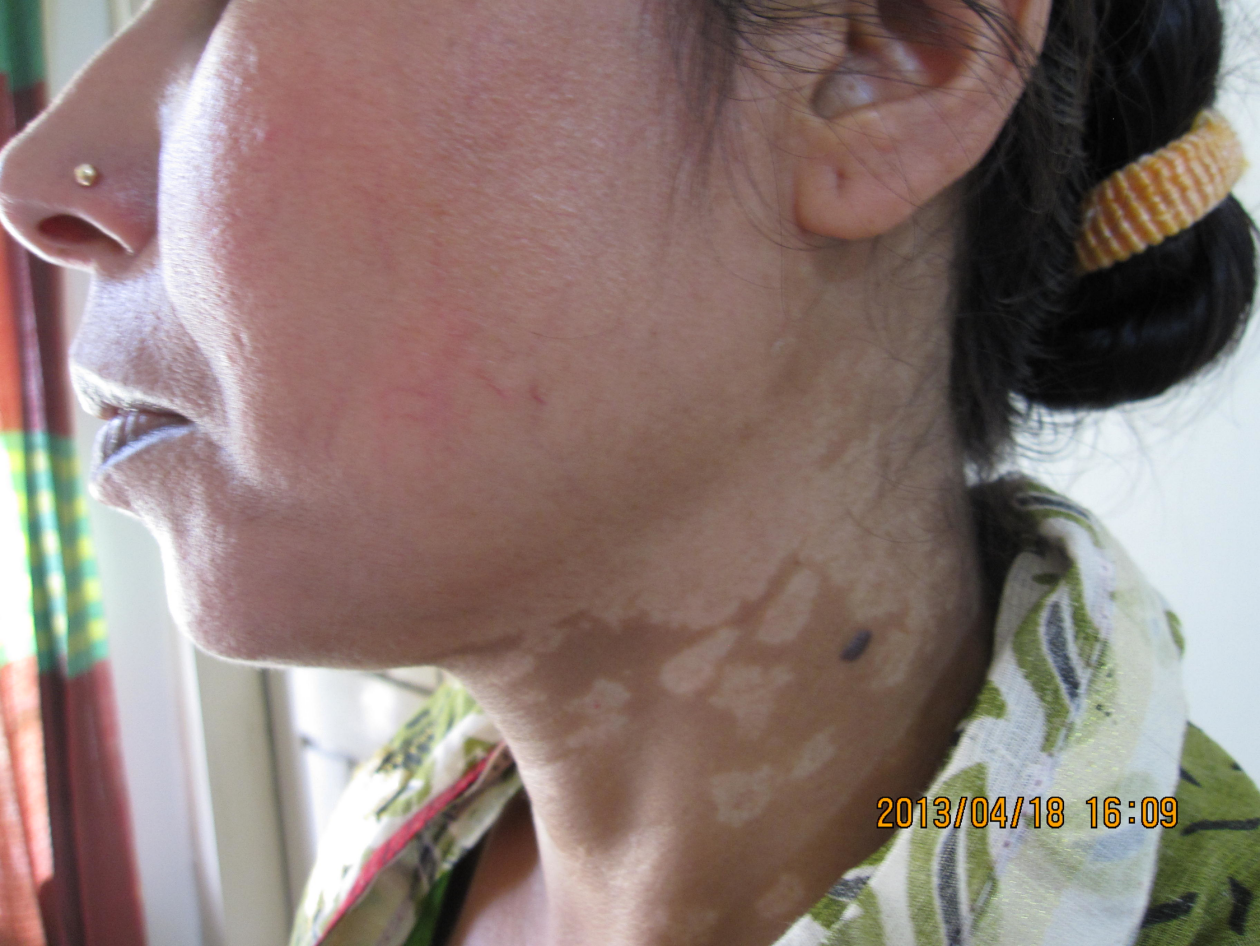
PKDL from Bangladesh: confluent macular rash involving most of the face (courtesy of Dr. Dinesh Mondal). PKDL; post-kala-azar dermal leishmaniasis.

**Fig 3:**
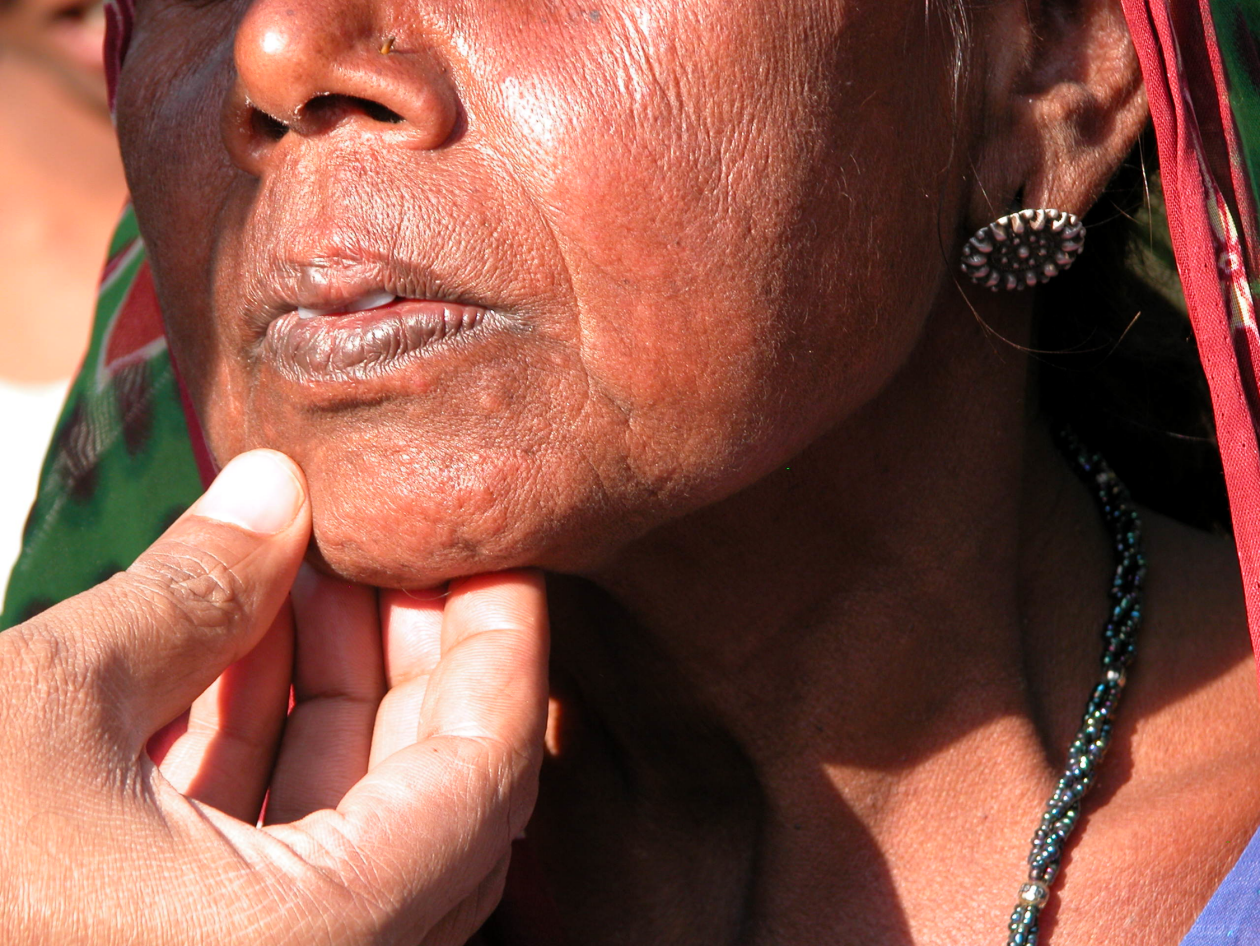
PKDL from India: discrete papules and infiltration of the chin, resulting in a plaque. PKDL, post-kala-azar dermal leishmaniasis.

**Fig 4:**
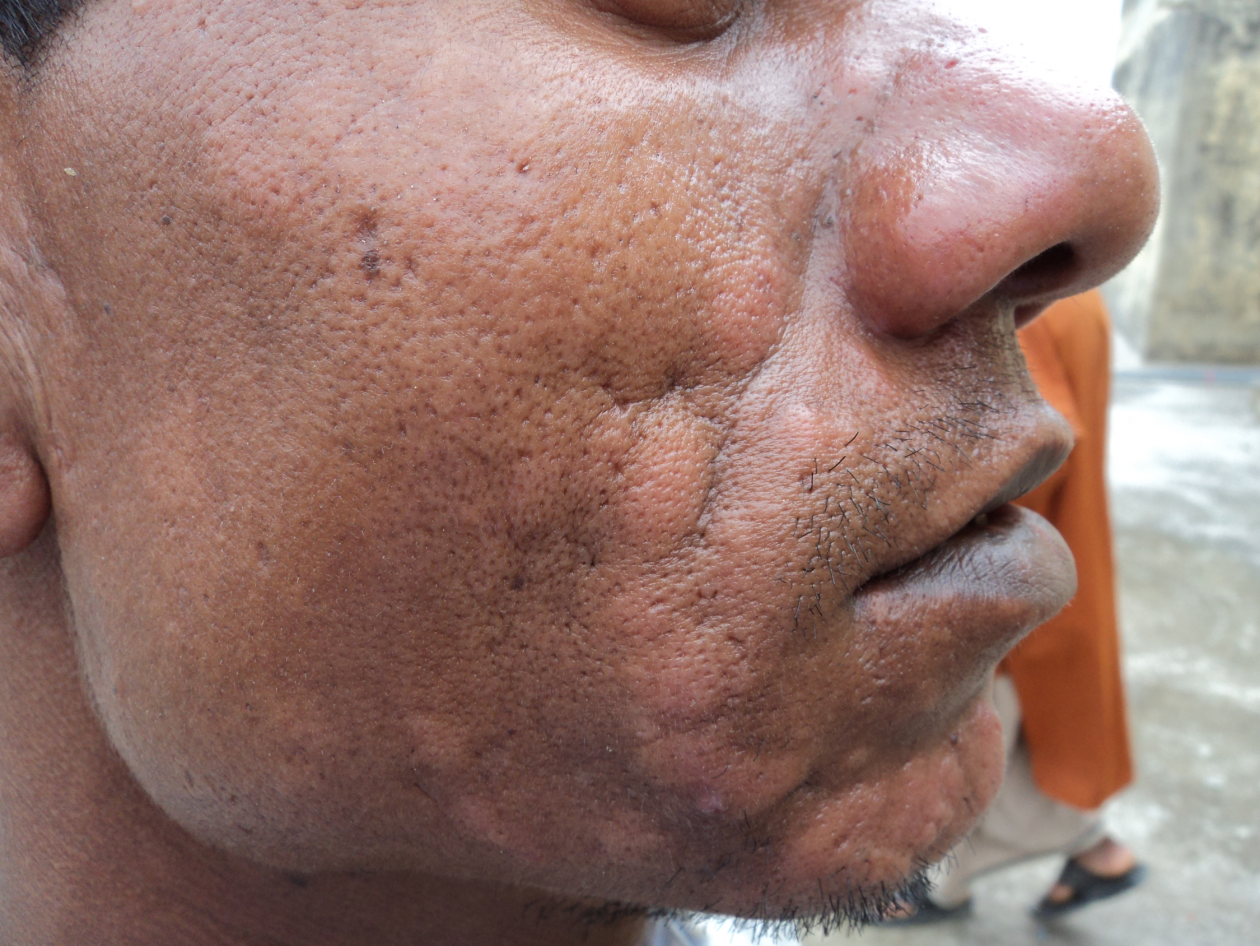
PKDL from Bangladesh: PN rash with infiltration of the cheek and chin. PKDL, post-kala-azar dermal leishmaniasis; PN, papulonodular.

An atlas on the clinical presentation and differential diagnosis of PKDL has been published [48]. In field studies, macular lesions, which can spread to involve most parts of the body, are most common. In hospital-based studies, often cases with a long duration of PKDL are reported with extensive papular, nodular, or mixed presentations; special forms include erythrodermic, warty, lupoid, and tumour-like forms (Figs 2–4).

The main differential diagnosis is with leprosy and vitiligo, but many other skin conditions can be mistaken for PKDL and vice versa, thus complicating case finding and management [48].

A clinical algorithm for diagnosis under field conditions has been proposed, but confirmation is often necessary [49].

Parasitological diagnosis can be done by slit-skin smear or by biopsy. In slit-skin smears, macular lesions will often fail to show parasites, while PN lesions will be positive in 20%–40% [13,50]. The yield is higher in a biopsy (imprint smears): 91% of PN lesions and 40% of macular lesions will show parasites [12]. Culture and immunohistochemical (IHC) staining improve routine haematoxylin–eosin staining [27].

Serological tests in the blood, such as the direct agglutination test (DAT) and rK39 RDT, may be used for screening; it is not clear whether antibodies may persist from previous VL or indicate the diagnosis of PKDL [10,51]. rK39 RDT can also be done on material from a slit-skin smear with 100% sensitivity and specificity [52].

Recently, serum adenosine deaminase (ADA) levels were found to be raised in PKDL and decreased during treatment [53].

PCR, nested PCR, and RLFP have all been demonstrated to be sensitive and can be done in material obtained by a slit-skin smear [10,16,54,55]. Quantitative PCR (qPCR) is sensitive (96%–100%) and allows monitoring of parasite reduction as a result of treatment [13,56,57]. New readouts such as loop-mediated isothermal amplification (LAMP) may be more appropriate for use in the field; recently, an adaptation using a closed-loop assay potentially reduced contamination and also showed potential as a test of cure in PKDL [52,58]. In contrast to degraded DNA, the presence of parasite DNA that can be amplified indicates live parasites.

## Treatment

Recommendations for treatment of PKDL have been published in WHO guidelines [59].

Currently, in the ISC, PKDL cases are treated according to national guidelines with miltefosine for 12 weeks; this regimen (50 mg twice per day) has a cure rate of 78% (intention-to-treat [ITT] analysis) and 93% (per protocol [PP] analysis). Shorter administration for 8 weeks leads to lower cure rates, while a higher daily dose (50 mg 3 times daily) or longer duration (16 weeks) showed higher cure rates of 96%–100%, although at the expense of increased gastrointestinal side effects [60–63].

As in VL, increasing resistance to miltefosine has also been reported in PKDL [10,64]. Parasites in pretreatment PKDL patients show more tolerance to miltefosine than VL samples [65]. Tolerance increases further after treatment of PKDL [66]. A higher parasite load pretreatment is a risk factor for relapse [66,67]. Relapses have also been described after paromomycin treatment [9].

In the Médecins sans Frontières (MSF) programme at Fulbaria, over 1,400 patients with clinically diagnosed PKDL were treated on an outpatient basis with 6 infusions of 5 mg/kg AmBisome administered over 3 weeks (total dose 30 mg/kg). Ninety-six percent of patients had macular lesions. Complete recovery of nodular and papular lesions and a complete or significant repigmentation of macular lesions were observed in 86.5% of patients at 12-month follow-up. Concerns were raised regarding potential complications with rhabdomyolysis [68]. A subsequent prospective cohort study on 110 patients (of whom 97% had macular lesions), using the same regimen to better categorise the safety profile of the regimen, resulted in 6 patients developing severe hypokalaemia during treatment; however, none were symptomatic nor developed rhabdomyolysis. Of 88 patients completing 12-month follow-up, 59 (80%) showed substantial or complete cure. In contrast, the same AmBisome regimen had similar efficacy outcomes (*n* = 161) but did not lead to severe hypokalaemia in India (personal communication, Koert Ritmeijer, MSF, Amsterdam, the Netherlands, to Eduard Zijlstra). Repeated courses (20 infusions for 3 courses) of conventional amphotericin B have been shown to produce good cure rates (>90%), but nephrotoxicity remains a problem [69].

Immunochemotherapy has been explored in Sudan; SSG for 40 days with alum-precipitated autoclaved *L*. *major* plus BCG vaccine or placebo showed better cure rates in the vaccine arm at 87% versus 53% cure. It was noted that leishmanin skin test (LST) conversion was a good surrogate marker for cure [70]. A third-generation vaccine developed to induce cluster of differentiation (CD)8^+^ T cells is expected to undergo study in the treatment of PKDL in Sudan in the near future [71].

Recurrence of VL after PKDL in non-immunocompromised patients is uncommon and was estimated to occur in 1 out of 700 cases [72].

### Assessment of cure

While the parasite load in PKDL is lower than in VL, a long duration of antileishmanial therapy is often given because there is no conclusive laboratory or clinical marker other than the disappearance of the lesions. In the case of macular lesions, this may take 12–18 months [10,12]. While in histological specimens after 3 months of treatment (SSG 10 mg/kg/day for 90 days) parasites can no longer be detected by microscopy, the mononuclear cell infiltrate still persists [12]. Both LAMP and qPCR could be useful, and the latter allows the comparison of the parasite (load) before and after treatment [52].

As in VL, serological tests are not useful for assessing cure; in 1 study, the DAT positivity rate decreased from 75% to 66% after treatment [12].

## Discussion

Current tools, research gaps, and needs for PKDL control are summarised in Table 2.

**Table 2:** PKDL—Summary of current tools and needs for the KAEP.

Intervention	Tool	In place	Research gap	Need
**Transmission**	• xenodiagnosis		• PKDL: who should be treated: 1. acute versus chronic 2. macular versus papulonodular 3. limited versus extensive • comparison of contribution of PKDL, VL, ex-VL, asymptomatics, HIV–VL • quantitative parasite load (qPCR) for infectivity	• xenodiagnosis studies to quantify the risk of transmission
**Screening and Dx** **biomarker**	• clinical Dx • rK39 RDT • PCR	• WHO self-learning course • WHO Manual for Health Workers • clinical Dx and rK39 RDT	• validated clinical algorithm • sensitive and specific serological test • biomarker for cure - PCR field application - immunological marker	• teaching of health workers • validation of clinical algorithm • point-of-care test • studies for biomarker
**Treatment**	• drugs: MF, AMB • immunomodulator	• universal policy for treatment with MF	• efficacy of current MF regimen (incl. resistance) • optimal regimen of AMB • alternative oral drug(s) • development of immunomodulator • drug penetration in the skin	• surveillance for resistance • safe, short course, oral drugs • candidate immunomodulator • PK studies with skin biopsies
**Prevention** • **by detection of VL and PKDL** • **by optimal VL treatment** • **by bed nets** • **by vector control**	• case finding • mono or combo treatment • LLINs • IRS	• active case finding for VL • single-dose AMB • LLIN distribution • IRS	• sustainability • PKDL rate for each VL treatment regimen • understanding of underlying immune responses • possible mode of action of immune modulator • long-term studies on efficacy, acceptability • efficacy of current intervention with DDT • efficacy of other insecticides	• political commitment • immunochemotherapy studies • alternative insecticides • other control measures

Abbreviations: AMB, AmBisome; Dx, diagnosis; IRS, indoor residual insecticide spraying; KAEP, Kala-azar Elimination Programme; LLIN, long-lasting insecticide-impregnated net; MF, miltefosine; PK, pharmacokinetics; PKDL, post-kala-azar dermal leishmaniasis; qPCR, quantitative PCR; RDT, rapid diagnostic test; rK39, recombinant K39; VL, visceral leishmaniasis.

### Importance of the immune response

The natural history of PKDL suggests that it is not a static but a dynamic condition in which the parasite load, onset and appearance of the clinical presentation, and tendency to self-heal vary with the developing immune response [22,73]. The roles of UV light and concurrent diseases such as malnutrition, tuberculosis, malaria, or reinfection are unclear and may prevent the establishment of a Th1 response or reverse an established Th1 response to a mixed Th1/Th2 response, thus possibly inducing an inflammatory response around ‘dormant’ parasites in the skin [74]. The role of repeated exposure to leishmanial infection is unclear, but it may also influence the immune responses. More information is needed on the leishmanial strains isolated from VL and PKDL patients in various presentations and the role (if any) of endosymbiotic infection.

### Prevention of PKDL

The best way to reduce the incidence of PKDL may very well lie in identifying the best treatment for VL that induces a strong and lasting Th1 response so as to prevent PKDL from developing. Currently, it is not known which drug (combination) has the lowest PKDL rate. The 13% PKDL rate after single dose AmBisome (the current VL regimen) is a reason for concern [3]. An immunomodulator that can supplement VL drug treatment should have priority. A candidate immunomodulator (CpG-D35) for cutaneous leishmaniasis that may also be useful in PKDL but is not expected to enter clinical studies in the near future is under development [75].

### Management of PKDL

More information is needed on the proportion of PKDL patients that self-cure, skin penetration and immunomodulatory effects of drugs used, and duration of treatment. The clinical cure of the lesions probably lags behind parasitological cure and subsequent immunological cure with the switch from a mixed Th1/Th2 response to a Th1 response (S1 Fig) [16]. A biomarker for cure is needed.

Short, ambulant treatment courses are needed, preferably with (an) oral drug(s). The current treatment with miltefosine is of long duration, and there are reports of increasing failure [13]. For any regimen, safety is of paramount importance because PKDL patients are mostly asymptomatic except for the rash. This is particularly important from an ethical point of view if patients are treated who otherwise may self-heal over time.

### Contribution to transmission

For the design of a cost-effective control strategy, the contribution of each category of PKDL patients should be studied: acute versus chronic, macular versus papular versus nodular, and limited versus extensive disease. In each clinical presentation, infectivity of the blood should also be studied. This can only be done by xenodiagnosis studies that quantify the potential for infectivity. For this purpose, insectaria have been established at Muzaffarpur (India) and Mymensingh (Bangladesh).

Clearly, this information is essential for the design and application of mathematical models in which the potential contribution of PKDL to transmission should be balanced and quantified against that of other clinical entities in the spectrum of VL: VL cases, ex-VL cases, HIV–VL coinfection, and asymptomatic individuals. Also, here, understanding the dynamics is important because the contribution of each may vary in the course of the condition as well as over time in the local epidemiological context [76,77]. On the same note, there is also confusion about cured VL and PKDL cases reentering the pool of susceptible individuals, while the current understanding supports that cure provides lasting immunity; however, continuing exposure seems necessary.

Other epidemiological information such as seasonality of sand flies and their behaviour, human migration, and the potential increase of HIV coinfection are among other factors to be explored. Dye and Wolpert recognised the influence of factors such as earthquakes, influenza, and malaria on the statistics of fever (VL or other) deaths, thus emphasising the dynamics over time [78].

In conclusion, the current optimal results obtained so far in the KAEP are fragile and must be consolidated before commitment is lost. History has shown that PKDL is a crucially important factor in the control of VL in the ISC. For the short term, a strategy for active case finding; the validation and implementation of diagnostic tools such as LAMP or qPCR; optimal treatment for VL aiming at the lowest possible PKDL rates; and the identification of short, safe, and effective treatment for PKDL are essential for the VL elimination efforts in the ISC to succeed and be sustained. For the long term, immunological studies and infectivity studies may provide insight in more targeted and more efficient approaches.

**Key learning points**

- The immune responses at various manifestations of PKDL need to be described (acute versus chronic, macular versus PN, limited versus extensive).
- The contribution of various manifestations of PKDL in transmission needs to be studied in xenodiagnosis studies and compared to the contribution of asymptomatics, VL patients, HIV-coinfected VL patients, and ex-VL patients, including parasite load by qPCR.
- Prevention of PKDL by optimal VL treatment is crucial.
- Optimal treatment for PKDL should be based on a safe oral drug with good skin penetration and immunomodulatory properties; it may include an immunomodulator.
- A point-of-care biomarker for cure needs to be identified to define the optimal duration of treatment for PKDL.

**Top 5 papers**

1. Banjara MR, Kroeger A, Huda MM, Kumar V, Gurung CK, et al. (2015) Feasibility of a combined camp approach for vector control together with active case detection of visceral leishmaniasis, post kala-azar dermal leishmaniasis, tuberculosis, leprosy and malaria in Bangladesh, India and Nepal: an exploratory study. Trans R Soc Trop Med Hyg 109: 408–415.
2. Hirve S, Boelaert M, Matlashewski G, Mondal D, Arana B, et al. (2016) Transmission Dynamics of Visceral Leishmaniasis in the Indian Subcontinent—A Systematic Literature Review. PLoS Negl Trop Dis 10: e0004896.
3. WHO. (2011) Regional Strategic Framework for Elimination of kala-azar from the South-east Asia Region (2011–2015). SEA-CD-239.
4. WHO. Post-kala-azar dermal leishmaniasis: a manual for case management and control. http://apps.who.int/iris/bitstream/10665/78608/1/9789241505215_eng.pdf (accessed March 23, 2016).
5. Zijlstra EE (2016) The immunology of post-kala-azar dermal leishmaniasis (PKDL). Parasit Vectors 9: 464

## Supporting information

S1 Table (DOCX)

S1 Fig (DOCX)
