# Supplementary material for: Post-kala-azar dermal leishmaniasis in the Indian subcontinent: A threat to the South-East Asia Region Kala-azar Elimination Programme
Source: PLoS Negl Trop Dis. 2017 Nov 16;11(11):e0005877. doi: 10.1371/journal.pntd.0005877 (PMC5689828; doi:10.1371/journal.pntd.0005877)
Supplement: S1 Table — (DOCX) [file pntd.0005877.s001.docx]

**S1 Table. Most important factors in the pathogenesis of PKDL (reviewed in ref [**[**45**](#_ENREF_42)**])**

| **Factors** | | **Main findings** |
| --- | --- | --- |
| **Host** | Immune responses | Cell-mediated immune (CMI) response  Profile   - Skin: Th2 response – persistence of IL-10, TGF-β - Systemically: Th1 response - IL-12, IFN-γ   Prominent role for   - Dendritic cells: damaged by UV light - T regulatory cells: inhibited by damaged dendritic cells - CD8 cells: various roles - cytotoxic, memory cells - Th17 cells: IL-17 and IL-23 upregulated, leading to elevated TNF-α and NO levels - Macrophages: polarized to M2 type   CMI stronger in acute PKDL (short interval after VL) and in the macular type  Humoral response   - High levels of antibody including IgG1 and IgG3 |
|  | Genetics | - Decreased function of the interferon-gamma receptor 1 gene (*IFNGR1*) linked to PKDL, not to VL. - In PKDL skin biopsies uniform low expression of IFN-γ and *IFNGR1*, possibly explaining the persistence of parasites |
|  | Previous treatment of VL | - Irregular dosage – higher PKDL rate - High dose, short course – lower PKDL rate |
|  | Drugs used for VL | AmBisome   - reduces IL-10 and TGF-β - more profound effect on reducing IL-10 and TGF-β than sodium stibogluconate (SSG)   Miltefosine   - stimulates T cells and macrophages *in vitro* - in human PKDL miltefosine modulates the cytokine response - increased levels of pro-inflammatory cytokines - decreased levels of anti-inflammatory cytokines - macrophages are activated |
| **Parasite** | Parasite species | - Mainly restricted to *L. donovani* endemic areas - *L. donovani* different in Africa vs Asia - Other Leishmania spp: PKDL usually only if immunocompromised host - Pathogenicity same as in VL |
|  | Antimony resistance | - Differences in polymorphism, differences in drug susceptibility, and differences in gene expression, possibly as a result of exposure to antileishmanial therapy - Antimony resistant *L. donovani* parasites have high potential *in vitro* to prevent SSG - induced dendritic cell activation and inhibit T regulatory cells through IL-10 |
|  | Endosymbiotic infection or superinfection | *Leptomonas seymouri* and *Parvibaculum lamentivorans* have been found   - suggesting a possible factor for development of PKDL |
