# Supplementary material for: Post-kala-azar dermal leishmaniasis in the Indian subcontinent: A threat to the South-East Asia Region Kala-azar Elimination Programme
Source: PLoS Negl Trop Dis. 2017 Nov 16;11(11):e0005877. doi: 10.1371/journal.pntd.0005877 (PMC5689828; doi:10.1371/journal.pntd.0005877)
Supplement: S1 Fig — (DOCX) [file pntd.0005877.s002.docx]

**S2 Figure**.

A simplified model showing the theoretical relationship between the occurrence of clinical cure, immunological cure (Th1 response) and parasitological cure after treatment in PKDL. The interval between these three outcomes and the duration of treatment is entirely hypothetical and may vary according to treatment given.

Parasitological cure

Immunological cure

Clinical cure

Treatment
